# Optimisation of the biological production of levulinic acid in a mixed microbial culture fed with synthetic grape pomace

**DOI:** 10.3389/fbioe.2024.1398110

**Published:** 2024-05-10

**Authors:** David Correa-Galetote, Antonio Serrano, Gustavo Ciudad, Fernanda Pinto-Ibieta

**Affiliations:** ^1^ Departamento de Microbiología, Facultad de Farmacia, Campus Universitario de Cartuja s/n, Universidad de Granada, Granada, Spain; ^2^ Instituto de Investigación del Agua, Universidad de Granada, Granada, Spain; ^3^ Departamento de Ingeniería Química, Facultad de Ingeniería y Ciencias, Universidad de La Frontera, Temuco, Chile; ^4^ Instituto del Medio Ambiente (IMA), Universidad de La Frontera, Temuco, Chile; ^5^ Centro de Excelencia en Investigación Biotecnologica aplicada al Ambiente (CIBAMA), Universidad de La Frontera, Temuco, Chile; ^6^ Departamento de Procesos Industriales, Facultad de Ingeniería, Universidad Católica de Temuco, Temuco, Chile

**Keywords:** biological production, feast and famine, grape pomace hydrolysate, mixed microbial culture, organic loading rate, polyhydroxyalkanoates

## Abstract

Levulinic acid (LA) is a polymer with a vast industrial application range and can be co-produced as a minor by-product during the biological production of polyhydroxyalkanoates (PHA). However, the influence of key parameters as tools for favouring the production of LA over PHA is still unclear. In this study, we investigated how several critical operational conditions, i.e., carbon-nitrogen ratio (C/N), organic loading rate (OLR) and airflow, can be optimised to favour LA accumulation over PHA production by a mixed microbial culture (MMC), using synthetic grape pomace (GP) hydrolysate as the substrate. The results showed that it was possible to direct the MMC towards LA accumulation instead of PHA. The maximum LA yield was 2.7 ± 0.2 g LA/(L·d) using a C/N of 35, an airflow of 5 L/min and an OLR of 4 g sCOD/(L·d). The OLR and, to a lesser extent, the C/N ratio were the main factors significantly and positively correlated with the biological synthesis of LA.

## 1 Introduction

Worldwide energy demand has doubled in the last 10 years (Sajid et al., 2021). This has resulted in a global primary energy consumption of around 160 × 10^18^ J per year, of which 80% is covered by fossil fuels (Di Bucchianico et al., 2022). However, excessive reliance on fossil fuels contributes significantly to greenhouse gas emissions, which in turn intensifies pollution in the environment and causes global warming (EPA, 2017). Hence, it is mandatory to mitigate petroleum-based consumption by searching for new, sustainable, green alternatives. Lignocellulosic biomass can be used as a substitute feedstock in producing biofuels and biochemicals, a strategy widely proposed within the transition plans for a sustainable economy and industry (Sherwood, 2020).

Among the different platform chemicals derived from biomass is levulinic acid (LA), also known as 4-oxopentanoic acid, a linear C5-alkyl carbon chain and considered one of the ‘Top 10’ value-added compounds in the world (Hayes and Becer, 2020). The estimated global market for LA is around US$ 22 million and is soon expected to increase enormously (Bazoti et al., 2023). Levulinic acid is a multipurpose platform chemical derived from biomass that can be used for synthesising a wide variety of potential biobased chemicals such as additives for fuels (methyl tetrahydrofuran and levulinate esters), biodegradable herbicides (delta-amino acid levulinic), resins and plasticisers (diphenolic acid) (Santiago and Guirardello, 2020; Charnnok and Laosiripojana, 2022). Nowadays, LA is usually chemically obtained from the acid hydrolysis of 5- and 6-carbon carbohydrates derived from cellulose at high pressures and temperatures, using waste and by-products of lignocellulosic biomass as raw materials (Jeong et al., 2018). However, the energy needs associated with the high temperature and pressure levels of the process result in significant financial and environmental expenses for this type of LA production (Signoretto et al., 2019). Additionally, operating with these conditions can also generate contaminating residues, including corrosive and hard-to-recycle homogeneous catalysts (Corona et al., 2018).

Implementing the microbial biosynthesis of LA from renewable carbon sources would be advantageous as it would result in lower downstream costs and reduce potential environmental impacts. The development of biotechnology tools has opened avenues to the design of new-to-nature pathways, enabling the bioproduction of LA by assembling combinations of enzymes not previously observed *in vivo*. The direct biological production of LA was achieved by the pioneering work of Zanghellini (2016), who used engineered *S. cerevisiae* and *Pichia stipe* enzymes. Likewise, Vila-Santa et al. (2021) identified several microbial precursors for the bioproduction of LA, proposing the transformation of D-alanine to LA via 2,5-Diaminovaleric acid by *Escherichia coli* and *Saccharomyces cerevisiae.* This proposed pathway is promising due to the low number of required metabolic steps and the high internal pools of D-alanine, which could be successfully improved in *E coli* and *S. cerevisiae*. However, a complete microbial pathway to produce LA as a product or an intermediate by a single microorganism has not yet been described. In contrast, the microbial LA metabolic pathway and its related genes have largely been deciphered, highlighting the transformation of LA into 3-hydroxyvalerate-coenzyme A by the sequential action of the enzymes LvaE, LvaD, LvaAB and LvaC, encoded by the *lvaABCDEFG* operon, followed by a β-oxidation of the hydroxyvalerate-coenzyme A to propionyl-CoA and acetyl-CoA in *Pseudomonas putida* KT2440 (Habe et al., 2020).

The operation with a mixed microbial culture (MMC) has been widely described as an interesting approach for the microbial-mediated biosynthesis of petroleum-based polymers due to the lower operating costs, easy adaptation to the use of agro-industrial waste as a carbon source and an enhancement of the robustness to changes in operating conditions (Montiel-Jarillo et al., 2017; Huang et al., 2018; Mohamad Fauzi et al., 2019; Wu et al., 2023). Regarding LA biosynthesis, it has been hypothesised that the beneficial synergistic cooperation between strains in the MMC could result in the best-performing pathway to produce LA from renewable sources (Vila-Santa et al., 2021). The use of the MMC has widely been oriented to PHA production from fermented waste, using sequential batch reactors (SBRs) subjected to the feast and famine (F/F) culture strategy (Fra-Vázquez et al., 2018; Correa-Galeote et al., 2022a; Gottardo et al., 2022). However, beyond PHA, the use of non-fermented waste in the MMC under the F/F culture strategy could lead to the accumulation of other compounds such as triacylglycerol, polyglucose or LA (Freches and Lemos, 2017; Pinto-Ibieta et al., 2020; Pinto-Ibieta et al., 2021; Argiz and Correa-galeote, 2022; Correa-Galeote et al., 2022b).

To the best of our knowledge, Pinto-Ibieta et al. (2020), Pinto-Ibieta et al. (2023) were the only authors who have reported the co-production of LA and other added-value compounds, mainly polyhydroxybutyrate and adipic acid, in an MMC subjected to the F/F culture strategy, using synthetic hydrolysed hemicellulose and xylose-rich substrate as carbon sources. The operational conditions that favour the specific accumulation of a particular target compound are still unclear. Specifically, although the results described by Pinto-Ibieta et al. (2020), Pinto-Ibieta et al. (2021), Pinto-Ibieta et al. (2023) show a high relative capacity of LA accumulation by the MMC using pentoses as carbon sources (maximum accumulation of 37% w/w), the operational conditions that could improve LA production have not been well established.

Therefore, other non-fermented agro-industrial waste should be tested to identify the feasibility of using other monosaccharides (hexoses) as carbon sources to feed an LA-producing MMC. Grape pomace (GP) is an agro-industrial waste from the winery sector that could be an attractive substrate for the bioproduction of LA since it contains up to 38% w/w (dry matter) of soluble carbohydrates (Corbin et al., 2015). As GP is produced at a rate of 0.13 tonnes per tonne of wine grapes, it is necessary to implement a suitable management method for its valorisation (Croxatto-Vega et al., 2019). Hence, the bioproduction of LA by using an MMC fed with GP could be an attractive waste management due to the high industrial potential and economic value of this chemical platform, as the estimated value of the LA global market is predicted to reach US$ 30 million in 2027 (Bazoti et al., 2023). Given the potential interest in developing a biological process for the synthesis of LA, it is mandatory to identify the operational conditions that favour the specific bioproduction of LA by an MMC using the F/F culture strategy. In this context, the main scientific novelty of this research is based on the effect of varying the key operational conditions, i.e., carbon-nitrogen ratio (C/N), organic loading rate (OLR) and airflow, to produce LA using an MMC at the F/F culture strategy with carbohydrates (composed of glucose and xylose) obtained from hydrolysed GP.

## 2 Materials and methods

### 2.1 Inoculum and synthetic carbon source obtention

Aerobic sludge was taken from the wastewater treatment plant in Temuco (Aguas Araucanía, Chile). We collected 10 L of aerobic sludge from the recirculation line on the aerobic reactor and let it settle for 12 h. In addition, 1.5 L of concentrated aerobic sludge was taken and used as inoculum. To ensure the reproducibility of the experiments, a synthetic GP hydrolysate was prepared based on the sugar composition previously reported by Serrano et al. (2023), i.e., 64% glucose, 31% xylose and 5% arabinose. Using a synthetic mixture made it possible to circumvent undesirable interferences in the evaluation of the suitability of the sugar mixture as a substrate for LA accumulation. Such interferences arise due to matrix effects from minor compounds in the grape hydrolysate, such as phenols or terpenes.

### 2.2 SBR set-up and operation

Fifteen 1-L working volume sequential batch reactors (SBRs) were operated in parallel to obtain an MMC enriched in LA-producing microorganisms. For that purpose, the SBRs were operated to achieve a pseudo-stationary state regarding the volatile solids (VS) concentration and LA production for at least five consecutive cycles. The SBRs were initially inoculated with 3.6 g VS/L of aerobic sludge. Each SBR was automatically fed and purged using a peristaltic pump connected to a compact DAQ system (cDAQ-9178 chassis, National Instruments, Austin, TX, United States) and a routine programmed using the LabView software (National Instruments). The SBRs were operated at 25°C in 24-h cycles, feeding each cycle with 200 mL of synthetic GP enriched with micro- and macronutrients (Pinto-Ibieta et al., 2020). Once the steady state had been reached, the dissolved oxygen (DO) was monitored to corroborate the SBR operation by feast/famine culture regimen. An example of the DO variation in relation to the differences in oxygen consumption during the feast and famine phases within the feeding cycles is shown in the Supplementary Material (Supplementary Figure S1). The pH was monitored throughout the experimental time, varying in a short range between 5.8 and 6.2 (Supplementary Figure S2). No pH adjustments were required. Samples were taken at the end of each two to three cycles to evaluate the processes.

### 2.3 Experimental design and statistical analysis

A 2^3^ factorial design was used with three factors, two levels, three replicates and five central points. A factorial design was used due to set-up limitations, since the factorial design enables the delineation of interactions among different factors with a lower number of runs compared to a full factorial design. The factors evaluated were OLR, airflow rate and C/N ratio. These factors and their respective levels were defined according to previous works employing an MMC subjected to the F/F culture strategy (Fang et al., 2019; Fra-Vázquez et al., 2018; Huang et al., 2016; Pinto-Ibieta et al., 2020; Pinto-Ibieta et al., 2021; Pokój et al., 2019). The matrix of the partial 2^3^ factorial experimental design is shown in Table 1. Pearson’s correlation analysis was performed using Sigmaplot^®^ version 11.0 on the different operational conditions, i.e., C/N ratio, airflow and OLR, and the values of LA production yield and accumulation were obtained at each experimental condition.

**TABLE 1:** Matrix of the partial 2^3^ factorial experimental design.

	C/N ratio	Airflow (L/min)	OLR (g sCOD/(L·d))
R1	20	3	3
R2	35	1	2
R3	5	5	2
R4	5	1	4
R5	35	5	4

OLR, organic loading rate; sCOD, chemical oxygen demand.

### 2.4 Analytical methods

Substrate consumption was measured by determining the reducing sugar concentration in the filtered samples (0.22-μm pore size PVDF membrane, Merck). Reduced sugar was quantified using the dinitrosalicylic acid (DNS) reagent method (Zhang et al., 2012; Prasertsung et al., 2017). The absorbance of the final solution was measured at 540 nm, using a GENESYS 10 s spectrophotometer (Thermo Scientific, United States) and glucose as a standard reagent (Merck, Germany). The VS (used for biomass production quantification) and soluble chemical oxygen demand (sCOD) were quantified using a standard technique (AWWA, 2005). LA and PHB were quantified and identified according to the methodology described by Lappalainen and Dong (2019) and Serafim et al. (2024) after a washing process to ensure the absence of dissolved sugars that would interfere with the results. The washing process consisted of three washes of the biomass. For this, 3 mL of the sample was centrifuged for 10 min at 12,000 g, and the pellet obtained was resuspended to 3 mL with distillate water. Further, the lyophilisation of this sample was carried out. The lyophilised biomass was resuspended in 1 mL of acidified methanol (20% H_2_SO_4_) with 0.65 mg/mL of benzoic acid as an internal standard (Sigma Aldrich). One ml of chloroform was added to this mixture, and the solution was kept in a thermoblock at 100°C for 3.5 h. After cooling, 0.5 mL of water was used for extraction. The chloroform phase was collected, and molecular sieves (0.3 nm) were added for water removal. One ml of the obtained chloroform phase was finally injected in a GC/MS column (Clarus 600, PerkinElmer). The column used to determine PHA concentration was a DB-FFAP 30 m × 0.25 mm × 0.25 um (Agilent), while an ELITE 1701 30 m × 0.25 mm × 0.25 um was used to determine LA concentration (PerkinElmer). The calibration curve was obtained by injecting a series of standards at different concentrations of LA and polyhydroxybutyrate (PHB) (Sigma Aldrich), previously subjected to the described procedure. Substrate degradation was measured by determining the concentration of reducing sugars and acetate in the filtered samples (0.22-μm pore size PVDF membrane, Merck). To quantify the total reducing sugars, the dinitrosalicylic acid (DNS) reagent method was used (Zhang et al., 2012; Prasertsung et al., 2017). Acetate was determined by gas chromatography in a flame ionisation detector (Clarus 400, PerkinElmer) using a NukolTM capillary column (Sigma-Aldrich, Darmstadt, Germany). The VS and soluble chemical oxygen demand (SCOD) were quantified using a standard technique (AWWA, 2005).

## 3 Results and discussion

### 3.1 Stability of the SBRs under different F/F operational conditions

**FIGURE 1:**
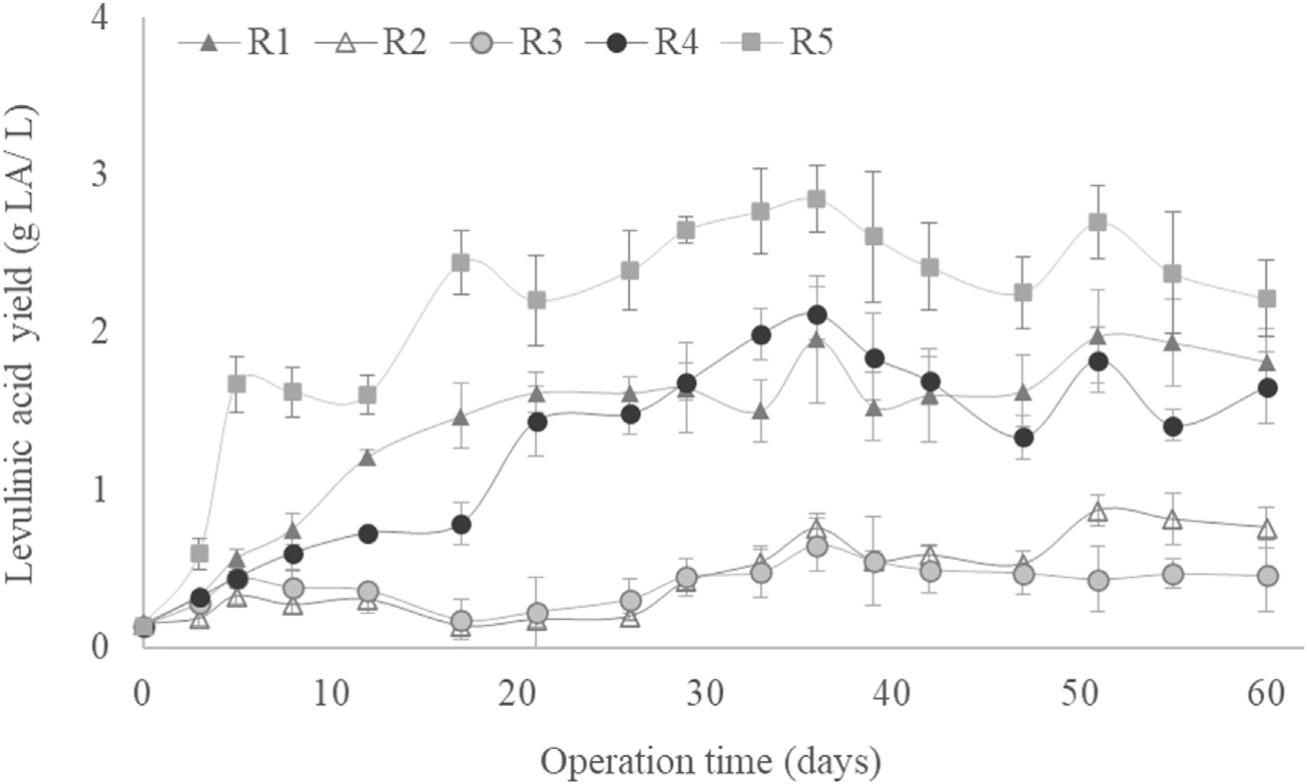
Volatile solids as a function of culture time for all operational conditions studied.

Figure 1 shows the VS variation as a function of culture time for the different experimental conditions described in Table 1. All SBRs started from 3.6 g VS/L and increased above 5 g VS/L throughout the operation time, except under R2 conditions, where the solids decreased up to an average value of 3 g VS/L. Thus, in both conditions, the biomass was completely removed from the SBR, indicating that the established MMCs could adapt and metabolise the fed substrate (Oliveira et al., 2017). The lower VS concentration reached under R2 conditions would be related to the necessity of adapting the MMC to these conditions, which would not be adequate for the initial microbial composition of the MMC (Jiménez-Páez et al., 2023). A similar VS decrease was observed in an SBR operated under the F/F strategy for the synthesis of LA by Pinto-Ibieta et al. (2020), who described a decline from 8.2 to 4 g VS/L when aerobic sludge was fed a carbon source composed of 80% xylose, 9% acetic acid, 6% furfural and 5% arabinose. The highest biomass production was achieved under R5 conditions (C/N ratio: 5, OLR: 4 g sCOD/(L·d) and airflow: 1 L/min), reaching a stable concentration of around 8.1 ± 0.77 g VS/L after 30 days of operation (Figure 1). This highest biomass production coincided with the SBR operated at the highest OLR. This was expected as low OLR conditions can lead to microbial growth limitation, whereas applying high OLR rates implies a higher carbon availability, resulting in increased biomass production (Alburqueque et al., 2010; Valentino et al., 2015). In a previous study, an increase in OLR from 1 to 7 g sCOD/(L·d) increased the biomass from 0.5 to 3.0 g VS/L in an MMC using acetate as a carbon source (Oliveira et al., 2017).

**FIGURE 2:**
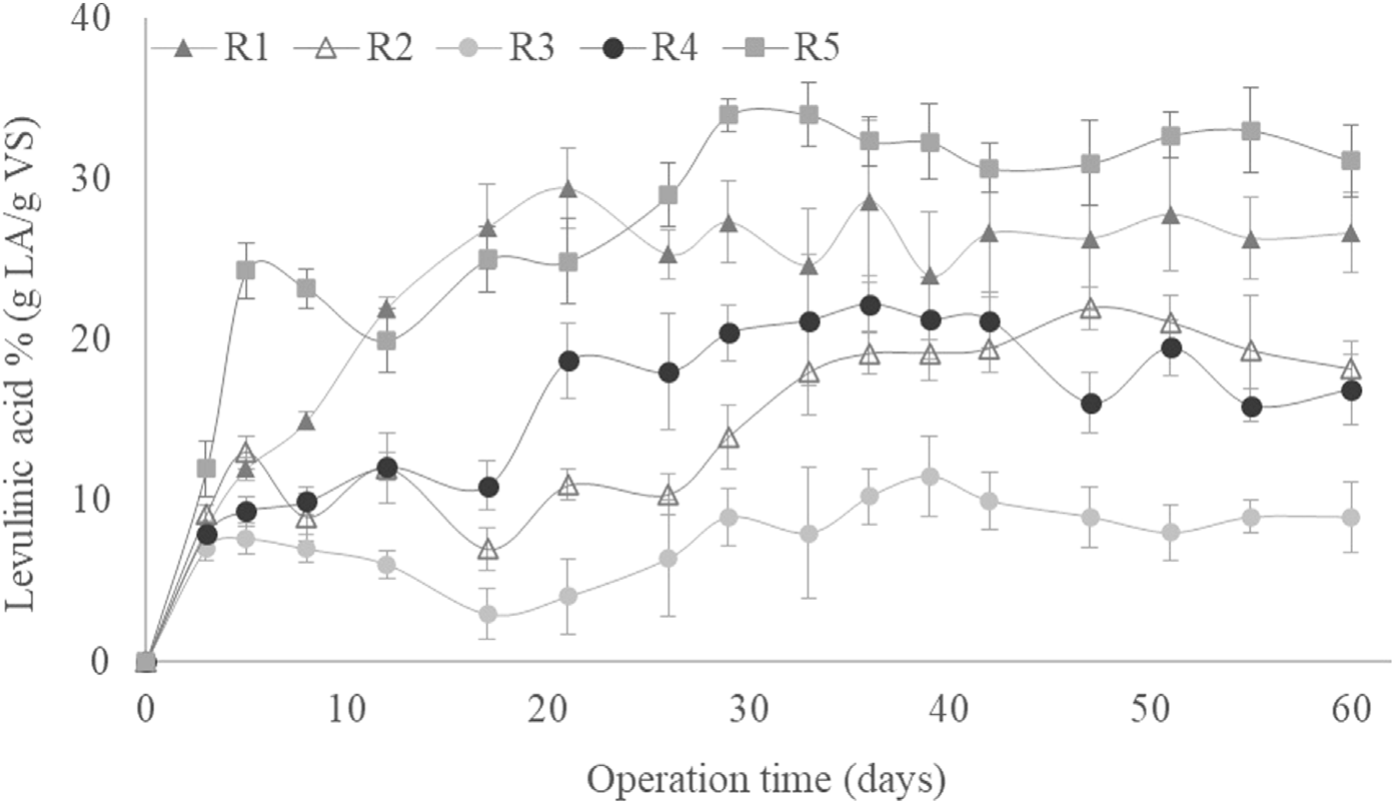
Reducing sugar concentrations at the beginning and end of the cycles as a function of the operating time during the pseudo-stationary stage.

The reducing sugar concentrations at the beginning and the end of each cycle operated under all SBR operational conditions throughout the 60 days of culture are shown in Figure 2. At the end of each cycle, there were no significant concentrations of reducing sugars, indicating that the synthetic GP was almost entirely consumed by the microorganisms in each cycle for all culture periods (Pinto-Ibieta et al., 2020). The high biodegradability of the used synthetic GP hydrolysate can be explained by its composition, which is rich in easily metabolised sugars (Section 2.1). The almost negligible concentration of reducing sugars at the end of each cycle indicates that the MMC could degrade all available substrate despite the difference in OLR (Figure 2). That also implies that there were no reducing sugars in the taken samples that would be chemically converted into LA during the experiment, thereby distorting the measured biological synthesis of LA (Kang et al., 2018).

### 3.2 Enrichment of the LA-accumulating mixed microbial culture under different operational conditions

**FIGURE 3:**
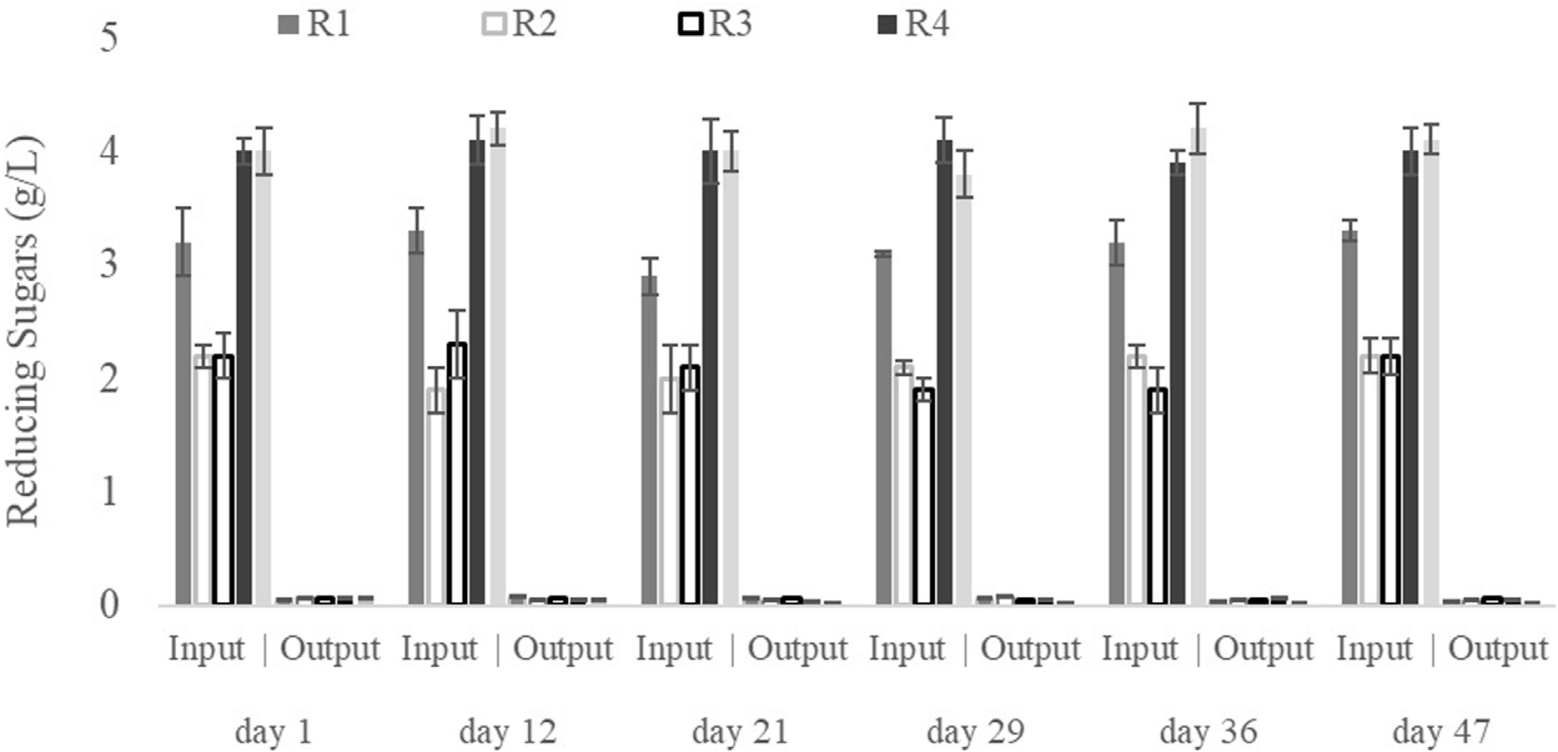
Levulinic acid production as a function of culture time for all operational conditions studied.

Figure 3 presents the LA accumulation (% w/w in dry weight) shown by the MMC throughout the experimental period under each set of conditions. The accumulation capacity was sufficient at the end of the experiment in all SBRs, although significant differences were found among the LA bioproduction for the different SBRs due to variations in the operational conditions. According to the biomass accumulation in the SBRs (Figure 1), the pseudo-stationary phase in LA accumulation was reached after 30 days of operation. The highest LA accumulation was found in R5 (using a C/N ratio of 35, airflow of 5 L/min and an OLR of 4 g sCOD/(L·d)), with an average LA accumulation of 35% (w/w) at the pseudo-stationary phase (Figure 3). A similar maximum LA accumulation value (37% (w/w)) was obtained by the MMC fed 2.5 g sCOD/(L·d) of hydrolysed hemicellulose composed mainly of xylose (Pinto-Ibieta et al., 2020). The LA accumulation in this work was always above 10% (w/w), regardless of the operational conditions (Figure 3). These values are higher than those obtained previously with SBRs for the bioproduction of high-added value compounds such as PHA using xylose or other waste as substrates (Huang et al., 2016; Yin et al., 2019; Correa-Galeote et al., 2022b). Hence, the sugar content from GP could be used as a suitable substrate for the biological production of LA, potentially converting this widely produced waste from wine manufacturing into a valuable by-product.

Strikingly, PHA production was not detected in any of the SBRs under the studied conditions, and the operational conditions and the carbon source imposed on the MMC did not permit the accumulation of PHA. Additionally, almost no acetic acid was accumulated in the SBRs during the experimental period. The absence of PHA and acetic acid indicates that the metabolic pathways carried out by the MMC were strongly directed to LA accumulation, preventing the transformation of the sugars from the substrates into volatile fatty acids, e.g., acetic acid for the subsequent biosynthesis of PHA, one of the main described metabolic route for PHA production (Vázquez-Fernández et al., 2022). Other authors using reducing sugars, such as glucose or xylose, obtained a selection of PHA-producing MMC (Cui et al., 2016; Huang et al., 2016; Yin et al., 2019; Li et al., 2022). The favour of LA over PHA would be an empirical demonstration of the biological synthesis of LA by the metabolic pathways proposed by Lee et al. (2019). Therefore, controlling the evaluated operational variables is crucial to stimulate the MMC to metabolise the reduced sugars from GP into LA instead of bioaccumulating PHA.

**FIGURE 4:**
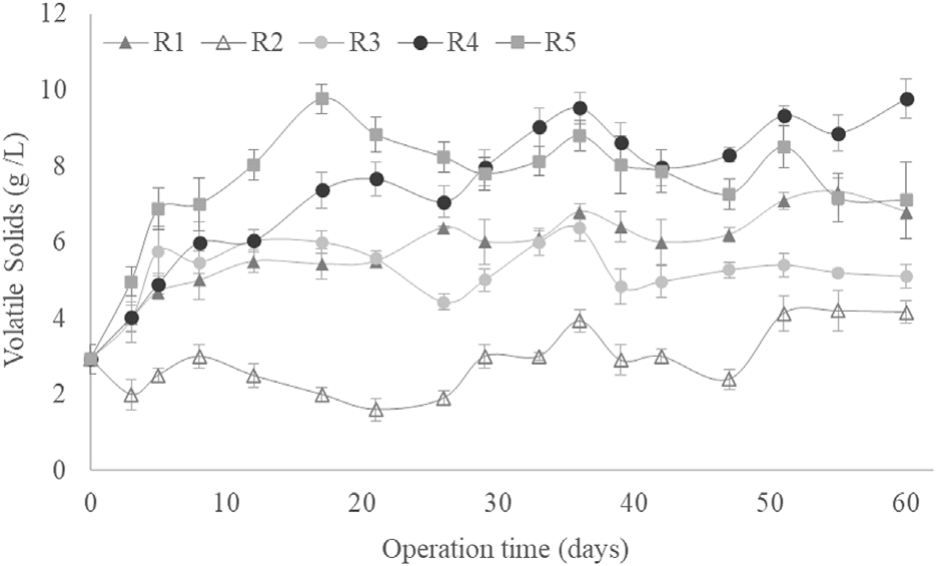
Levulinic acid yield as a function of culture time for all operational conditions studied.

The LA production yield values showed similar trends those achieved for LA accumulation (Figures 3, 4). Maximum values were obtained at the R5 conditions, i.e., an average value of 2.7 ± 0.2 g LA/L (2.01 g COD-LA/L) was obtained after 30 days of operation. This concentration of LA expressed as COD, represents a conversion rate of 54% with respect to the substrate added to the reactors. Like LA accumulation, the lowest LA production yield corresponded to R2 and R3 conditions, around 75% lower than those described for the R5 conditions (Figure 4). Despite this reduction, the LA production yields obtained under R2 and R3 conditions were similar to those described by Pinto-Ibieta et al. (2020), who reported an LA production yield of 0.9 g LA/L using a substrate containing 80% xylose, 9% acetic acid, 6% furfural and 5% arabinos, an F/F cycle of 12 h, an airflow of 4 L/min, an OLR of 2.2 g COD/(L·d) and a C/N ratio of 22.5. In this context, the higher LA production yield at R5 would result from the higher OLR and C/N ratio fixed by these authors.

### 3.3 Impacts of OLR, C/N ratio and airflow on LA accumulation capacity

The highest LA accumulation (% w/w) was obtained at conditions different to those that resulted in the highest achieved biomass (R4: C/N ratio of 5, airflow of 1 L/min and an OLR of 4 g sCOD/L). This suggests that the evaluated operational parameters could modulate the obtained LA yield in the SBRs (Figures 3, 4). Table 2 shows the values of Pearson’s correlation coefficients of the different operational parameters and the obtained values of LA production yield and accumulation. Based on these findings, the OLR was the main factor in both LA production yield and accumulation. The high and significant value of the Pearson’s coefficient observed between the OLR and the LA production yield indicates that a higher OLR would directly entail a higher LA production yield, albeit within the evaluated range. A positive correlation between PHA accumulation and OLR has previously been described by Simona et al. (2022) and Carvalho et al. (2014) for MMCs using a feeding solution made of a synthetic mixture of acetic and propionic acids and fermented molasses, respectively. Thus, the OLR could be a factor related to the accumulation capacity of an MMC. However, it is, most likely, not the only factor involved in promoting a specific pathway for LA accumulation instead of PHA.

**TABLE 2:** Pearson’s correlation coefficients of C/N ratio, airflow and OLR and the obtained values of LA production yield and LA accumulation.

	Airflow	OLR	LA production yield	LA accumulation
C/N ratio	0.000	0.000	0.344	0.653*
Airflow		0.000	0.240	0.124
OLR			0.890*	0.676*
LA production yield				0.896*

*p < 0.05; OLR, organic loading rate; LA, levulinic acid.

Likewise, LA accumulation was significantly and positively related to the C/N ratio (Table 2). Experimentally, the maximum LA accumulation, i.e., 35% (w/w), occurred when the OLR was 4 g sCOD/(L·d). The C/N ratio was 35/1, regardless of the airflow (1 or 5 L/min) (Figure 3), according to the observed Pearson´s correlation values (Table 2). According to the literature, a C/N ratio of 22.5/L facilitates a similar bioproduction (37% (w/w)) in an MMC fed with hemicellulose hydrolysate (Pinto-Ibieta et al., 2020). However, in another study, a higher C/N ratio of 30/L resulted in a low LA bioproduction; an accumulation of only 7% was achieved using hemicellulose as a carbon source (Pinto-Ibieta et al., 2023). Therefore, despite the observed influence of the C/N ratio on the LA accumulation capacity, the role of the C/N ratio in LA accumulation by the MMC is still unclear.

Contrary to the OLR and the C/N ratio, the evaluated range of airflow showed no influence on LA production yield and accumulation (Table 2). Different authors have reported that an airflow of 1 L/min in an MMC operated using F/F to produce PHA allowed to achieve a satisfactory accumulation level (Moralejo-Gárate et al., 2011; Moralejo-Gárate et al., 2012; Moralejo-Gárate et al., 2013; Moita et al., 2014; Mohamad Fauzi et al., 2019). Therefore, airflow may not greatly influence LA accumulation as long as it is not a limiting factor.

The obtained results demonstrate the feasibility of the biological synthesis of LA by an MMC using GP hydrolysate as substrate, highlighting the control of the OLR and C/N ratio as the crucial factors to enhance the microbial pathway that results in the intracellular accumulation of LA. Still, there is a shortage of information regarding the metabolic process, including the identification of the microorganisms involved and the empirical validation of the routes suggested in the literature. Further research in this regard would allow us to understand the process better and propose new improvement strategies that would result in a greater capacity for LA accumulation in these systems.

## 4 Conclusion

The present work demonstrates that the bioproduction of LA is possible by using an MMC fed with a mixture of reducing sugars (glucose, xylose and arabinose), which could be potentially obtained from sugar-rich agro-industrial waste such as GP. The OLR and, to a lesser extent, the C/N ratio were the main factors significantly and positively correlated with LA accumulation and production yield. These results pave the way for the biological synthesis of LA by microorganisms, supporting the use of LA as a metabolic intermediate in microbial metabolism. Based on our findings, it appears that the microbial consortia within the settled MMC can biosynthesise LA through a syntrophic pathway. This is significant because the *in vivo* generation of LA by a single microorganism has not been seen to date.

## Data Availability

The raw data supporting the conclusion of this article will be made available by the authors, without undue reservation.
