# Supplementary figures and images for: Optimisation of the biological production of levulinic acid in a mixed microbial culture fed with synthetic grape pomace
Source: Front Bioeng Biotechnol. 2024 May 10;12:1398110. doi: 10.3389/fbioe.2024.1398110 (PMC11116726; doi:10.3389/fbioe.2024.1398110)

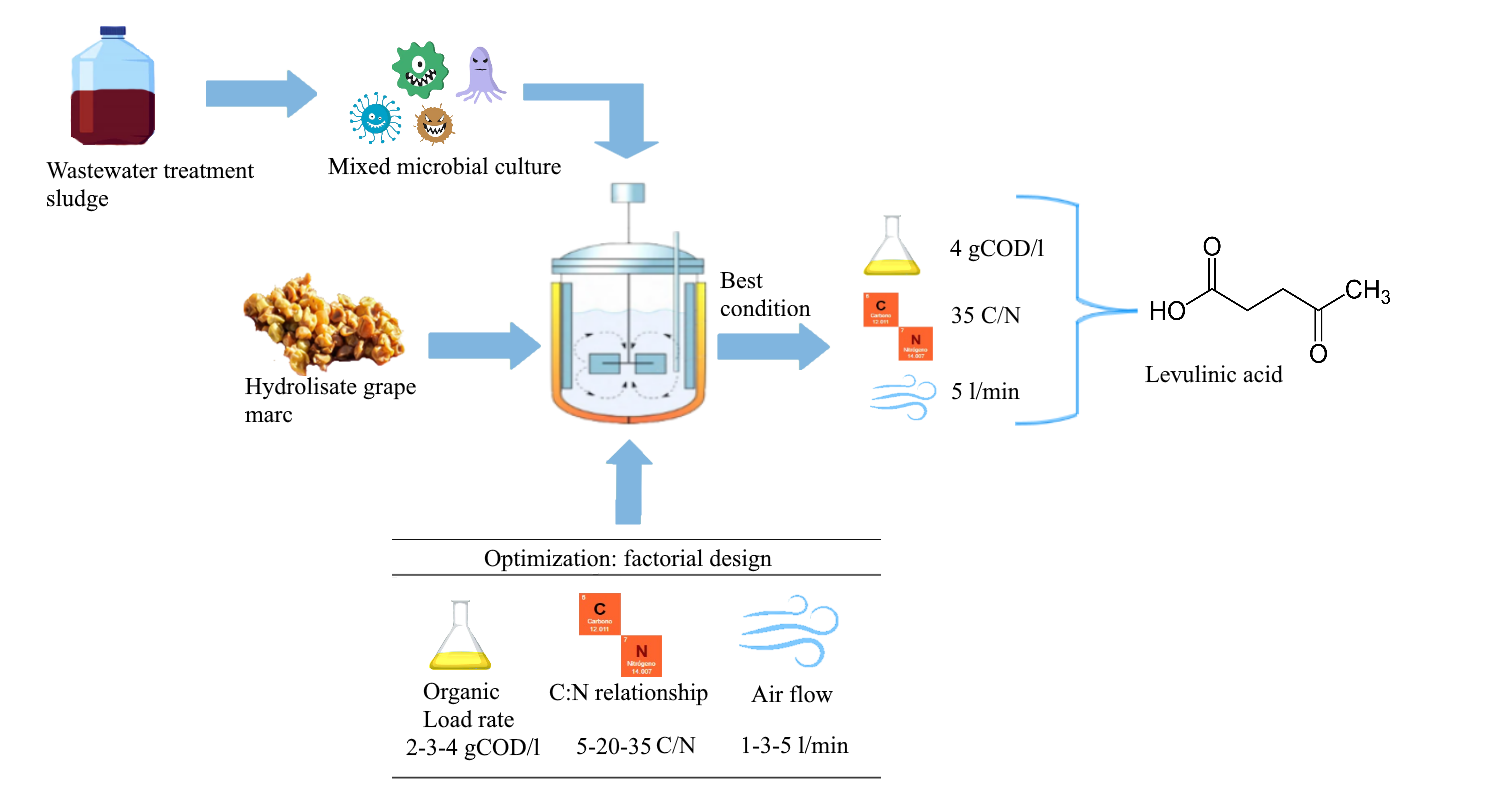

Supplement: Supplementary file 1 [file Image1.PNG]
