# Supplementary material for: Optimisation of the biological production of levulinic acid in a mixed microbial culture fed with synthetic grape pomace
Source: Front Bioeng Biotechnol. 2024 May 10;12:1398110. doi: 10.3389/fbioe.2024.1398110 (PMC11116726; doi:10.3389/fbioe.2024.1398110)
Supplement: Supplementary file 2 [file DataSheet1.docx]

**Supplementary material**

**Figure S1:** Example of the dissolved oxygen variation during the cycle of the day 50 for R5

**Figure S2.** pH evolution throughout the experimental time for all studied conditions
